# Sleep-Driven Computations in Speech Processing

**DOI:** 10.1371/journal.pone.0169538

**Published:** 2017-01-05

**Authors:** Rebecca L. A. Frost, Padraic Monaghan

**Affiliations:** Department of Psychology, Lancaster University, Lancaster, United Kingdom; Utrecht University, NETHERLANDS

## Abstract

Acquiring language requires segmenting speech into individual words, and abstracting over those words to discover grammatical structure. However, these tasks can be conflicting—on the one hand requiring memorisation of precise sequences that occur in speech, and on the other requiring a flexible reconstruction of these sequences to determine the grammar. Here, we examine whether speech segmentation and generalisation of grammar can occur simultaneously—with the conflicting requirements for these tasks being over-come by sleep-related consolidation. After exposure to an artificial language comprising words containing non-adjacent dependencies, participants underwent periods of consolidation involving either sleep or wake. Participants who slept before testing demonstrated a sustained boost to word learning and a short-term improvement to grammatical generalisation of the non-adjacencies, with improvements after sleep outweighing gains seen after an equal period of wake. Thus, we propose that sleep may facilitate processing for these conflicting tasks in language acquisition, but with enhanced benefits for speech segmentation.

## Introduction

Language acquisition is complex: learners must process information for multiple linguistic tasks quickly, simultaneously, and online, from the same transient input [1]. Two tasks that are critical for language proficiency are learning to identify words from continuous speech, and learning to process the grammatical rules that constrain the way those words can be used. There is broad agreement that segmenting speech into individual words relies (at least in part) on computing the statistics of the input [2, 3, 4]. In contrast, within the statistical learning literature there are divided opinions over the type of computation applied to grammatical processing [5, 6], where one view is that similar statistical operations apply to both segmentation and grammatical tasks [2, 7, 8, 9], and the alternative is that the operations are qualitatively distinct, with statistical processing for segmentation, but symbolic algebraic computations applying to grammatical tasks [10, 11, 12].

In either case, there are potentially conflicting requirements for these tasks, with segmentation requiring learning instances of lexical items from speech, and generalisation of structure necessitating abstraction away from these specific instances [13]. For example, one proposal for early language acquisition is that children initiate language learning through a process of instance-based learning, where whole frequently-occurring sequences are represented in the child’s language, and are only gradually broken down into their constituent parts to facilitate flexible use of the constituent words in a variety of contexts [5, 14]. Peña et al. provided behavioural evidence of a dissociation between speech segmentation and structural generalisation, and highlighted the potential difficulty faced by learners when requirements for these tasks conflict [12]. In their study, participants were trained on an artificial language comprising items that were defined by non-adjacent transitional probabilities, that is a 100% reliable pairing of two items, separated in speech by a statistically independent item. The language followed an AXC structure, where A, X, and C were distinct consonant-vowel syllables, and where A always predicted C, while X was free to vary over a set of three other syllables. After 10 minutes of exposure, learning was assessed through a two-alternative forced-choice (2AFC) task; participants were presented with pairs of items, and had to select which one from each pair best fit the language they heard during training (see the Materials section below for further details of the 2AFC test trials). Findings indicated that participants were able to segment the speech (demonstrated by their preference for words over part-words on a 2AFC segmentation task), but were unable to generalise the non-adjacencies to novel consistent forms (evidenced by their lack of preference for new grammatical items over part-words on a 2AFC generalisation task). It is important to note, though, that participants were able to generalise when items in the speech stream were separated by short pauses (but see references [15] and [16] for a discussion of the structures tested by the generalisation task), and recent research by Frost and Monaghan demonstrated that segmentation and grammatical generalisation can be accomplished simultaneously when the requirements for these tasks do not conflict [8].

Subsequent research has seen much deliberation about the processes underlying these tasks of speech segmentation and grammatical generalisation (e.g. [8, 15, 17, 18]), yet there is broad agreement that learners must accomplish both tasks in order to reach linguistic proficiency. The question remains, though, of how learners come to solve these tasks when their requirements conflict in the absence of additional cues—as is often the cause in natural language [5, 14]. One way of strengthening the memory representations of words and grammatical rules is through sleep-related consolidation, which could potentially support learning specific instances *and* abstraction of structure simultaneously through the separation of these processes during sleep [19]. This would mean that sleep may enable segmentation and generalisation to improve at the same time, but independently of one another.

Indeed, sleep has been shown to have a profound influence on improving memory for particular instances (see [20] for a review) as well as abstraction and generalisation across a range of tasks [21, 22, 23], with the benefit of sleep for learning resulting from initial representations of stimuli in the hippocampus being transferred to the neocortex [24, 25, 26]. Importantly, for statistical language learning, Nieuwenhuis, Folia, Forkstam, Jensen and Petersson [27] found that sleep encouraged participants to respond to the structure of statistically defined grammars, whereas staying awake resulted in responses based only on frequencies of subsequences in the language. Further, Gómez, Bootzin and Nadel [28] showed that infants exposed to a language containing non-adjacent dependencies (presented in segmented speech) were able to generalise the grammatical structure after sleep.

Recently, Mirković and Gaskell [29] demonstrated a dissociation between effects of sleep for learning specific instances versus learning regularities that were applied more broadly in the language for an artificial vocabulary learning task. They examined sleep-related consolidation of both arbitrary word-referent mappings and the systematic regularities that governed the way these mappings could be used in linguistic structures (i.e. the co-occurrence of root words with morphological markers that indicate syntactic information such as gender), and found that sleep may yield preferential consolidation of specific (rather than structural) information.

While we know that sleep may benefit both individual word learning (e.g., [29, 30, 31, 32, 33]) and generalisation of structure (e.g., [27, 28, 34]), sleep research has yet to test learners on their simultaneous processing of word-like items and rule-like structures presented in speech when those tasks are in conflict. Whereas Mirković and Gaskell tested learning of specific instances and generalisation over those instances when there was no conflict between these tasks [29], it is still unknown how sleep might affect learning when the requirements for these tasks do conflict, such as that found in progressing from learning of words or multi-word utterances toward discovering how those sequences can be divided and recombined for productive language generation (e.g., [5]).

In this study, we tested directly whether sleep-driven changes in statistical computations could be observed for conflicting tasks of speech segmentation and generalisation of structure, with the expectation that sleep may permit improvements on both tasks in the absence of changes to the speech signal itself (i.e., with no additional cues). There are several possibilities for how sleep may assist the acquisition of these two tasks. First, sleep may provide a general learning effect, supporting both tasks, in which case we would observe an improvement to both speech segmentation and grammatical generalisation. Second, if sleep particularly affected abstraction, with learning to segment being better served by remaining awake [27], then we would see a sleep-specific improvement that is specific to grammatical generalisation. A third alternative is that sleep affects segmentation but not generalisation, consistent with the role of sleep in strengthening memories for individual items (e.g., [29, 31, 32, 35]). In this case, the consolidation of individual lexical items from the speech stream could override flexible learning of the abstract structure, such that segmentation improvement interferes with learning to generalise (see [28], for similar argument). Should this be the case, then we would see a benefit of sleep for the segmentation task only.

A final possibility is that sleep effects for these tasks may not only differ in absolute terms (i.e., improvement versus no improvement), but may also be temporally distinct—such that they emerge and dissipate at different times. In particular, there may be an effect of the immediacy of sleep after learning [33], and different patterns of persistence of learning seen for each task over time. To address this, we included in our experimental design participant groups who slept soon after first exposure to the stimuli (*sleep-first* group), and compared performance to another group who slept after a prolonged interval following first exposure (*wake-first* group). We tested these groups both 12 and 24 hours after initial learning. If the time course of sleep-related learning is distinct for segmentation and generalisation, then we would expect to see different patterns of improvement for these tasks in the 12 and 24 hours following learning. If the time-course of sleep-related learning is the same for these tasks, then we would expect to see similar patterns of improvement for segmentation and generalisation.

## Method

### Participants

Participants were 72 students at Lancaster University (30 males, 42 females) with a mean age of 20.3 years (SD = 2.76), who received £11 or course credit. Sample size was determined from Nieuwenhuis et al.’s [27] study of sleep effects on grammar learning (33 sleep participants), but was divisible by the number of conditions, with 36 participants in the sleep- and wake-first conditions (data collection was stopped when this number was reached). Data for an additional four participants were removed from the analysis for technical reasons (N = 1) and for failing to comply with experimental instructions (taking daytime naps, N = 3). All participants were native-English-speakers, with no known history of sleep or language disorder. Participants were required to avoid drinking alcohol between experimental sessions, and refrain from consuming caffeinated products in the two hours leading up to each session. This was verified by a questionnaire. The study was approved by Lancaster University Department of Psychology Ethics Board, and carried out in accordance with the provisions of the World Medical Association Declaration of Helsinki. All participants gave written consent upon arriving for their first session.

Participants completed a sleep diary for four days prior to the experiment, which was an adaptation of the NHS standard sleep diary [36]. Participants in the sleep-first condition reported an average of 7.55 hours (SD = 1.13) sleep per night, and participants in the wake-first condition averaged 7.44 hours (SD = .93) sleep per night during this four-day period. For the night of sleep that intervened experimental sessions, actigraphy data was confirmed by the self-report data.

### Materials

#### Training stimuli

Speech was produced using the Festival speech synthesiser [37]. The experiment used the same nine syllables as in Peña et al.’s study (/pu, ki, bε, du, tæ, gæ, li, ræ, fəʊ/) [12], plus nine additional syllables (/weI, vaʊ, sɔ, zeI, ʃəʊ, maʊ, nɔ, ʒeI, hi/), with each syllable lasting approximately 230ms. The structure of the language was based on that used by Peña et al., where non-adjacent dependencies defined both the words and the grammatical structure of the language [12]. Each word comprised three syllables and followed an AXC structure, where each A exactly predicted each C, but X varied over a set of syllables. There were three non-adjacent pairings (A_1_XC_1_, A_2_XC_2_, and A_3_XC_3_).

Gómez [38] found that amount of variation in the X position in pre-segmented sequences affected learning of non-adjacencies: specifically, a set size of 24 yielded learning, whereas smaller set sizes did not. While it might have been informative for us to include this larger set size, we opted to use 3X, 6X and 12X to reduce the likelihood that participants would perform at ceiling level (given the nature of the study, it was particularly important that we leave room for consolidation). We therefore varied whether participants heard 3, 6, or 12 syllables in the X position, which occurred with equal frequency within the non-adjacencies during training. For the 3X language, transitional probabilities between all adjacent syllables were .33. For the 6X language, transitional probability from an A to an X syllable was 0.167, but transitional probabilities from X to C, and from C to A were .33. For the 12X language, transitional probabilities were .33, apart from the A to X transition, which was .083.

To control for possible preferences for certain syllables in certain positions, and *a priori* preferences for particular dependencies between syllables, we constructed eight different versions of the language with pseudo-random assignment of syllables to positions within the words—subject to the constraint that syllables beginning with plosives occurred in A and C positions, and syllables beginning with continuants appearing in the X position (as was the case in [12]). These versions were counterbalanced across participants.

For the training speech, a 10.5-minute-long continuous stream of synthetic speech was generated by concatenating AXC strings, all of which were presented an equal number of times. Speech streams faded in and out for the first and last 5 seconds respectively, to prevent information from speech onset and offset from influencing performance.

#### Testing stimuli

A two-minute-long continuous speech stream was created in the same way as the training speech, which was presented at the start of each testing session. As short (2 minutes) versus longer (10 minutes) exposures have not been found to qualitatively change processing of these types of speech stream ([12], Experiment 5), this enabled us to examine the manner in which sleep affected subsequent processing for segmentation and generalisation of the refreshed speech, as distinct from effects that are due only to memory consolidation affected by sleep.

We constructed tests of segmentation and generalisation similar to those used by Peña et al. [12]. For the segmentation test, we constructed 18 part-words for comparison against words, which occurred in the speech but did not reflect the non-adjacent dependency structure of the language. Nine part-words each of the form XCA and CAX were used. Test pairs were constructed by matching each part-word with its corresponding word (so an A_1_X_2_C_1_ item was paired with an X_2_C_1_A_1_ part-word).

Generalisation test-pairs consisted of part-word vs. rule-word comparisons. Rule-words were items that had not appeared in the familiarisation stream, but were congruent with the grammatical structure of the language. Nine rule-words were created by substituting the X syllable from an AXC item with an A or C syllable from another item in the language that never occurred in that position during familiarisation. The nine rule-words each appeared twice; once with an XCA part word, and once with a CAX part-word.

Training and testing stimuli were presented using a laptop computer, and speech was delivered at a comfortable volume via closed-cup headphones.

### Procedure

Participants were randomly allocated to one of two consolidation conditions (sleep-first, and wake-first), and within these conditions 12 participants were each assigned to the 3X, 6X, and 12X languages.

**Fig 1:**
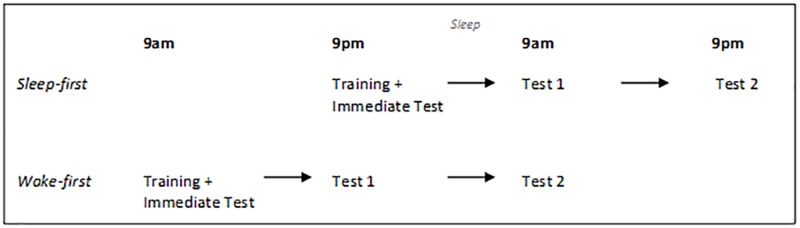
Testing times for the sleep-first and wake-first conditions.

Training occurred at 9pm for the sleep-first condition, and at 9am for the wake-first condition. Subsequent sessions took place 12 and 24 hours after the initial visit (see Fig 1). We opted to include a full night of sleep into the paradigm as the stages of sleep implicated in improvement on these tasks (REM and SWS) are typically captured in over-night sleep, but not always in short day-time naps.

For the training session, participants were instructed to pay attention to the language and think about possible words it may contain. Participants then heard the 10.5-minute familiarisation stream, and this was immediately followed by a test to establish baseline performance. During testing, participants were instructed to select which of two items best matched the language they had just heard, responding via key press with “1” for the first or “2” for the second sequence. For each of the 36 test trials, participants heard a word and a part-word (segmentation trials), or a rule-word and a part-word (generalisation trials). The presentation order of test-pairs and the position of the correct response within each pair was randomised. The same test items were used across all set-sizes.

Following training, participants underwent a 12-hour consolidation period containing either sleep or wake before returning for their second session. They attended their final session a further 12 hours later. For each subsequent test, participants returned to the laboratory, where they were presented with the refresher speech stream. They then completed the same test as in the first session, comprising all 36 test-pairs. All sessions took place in the same room, and participants were tested individually in an isolated booth.

In the evening sessions, participants were given a wrist-watch style Actigraph^™^ sleep monitor to wear overnight. Sleep length for each participant was scored using Actilife software [39]. Sleep length for the sleep-first condition was 6.03 hours between training and test 1, and was 6.25 hours between test 1 and test 2 for the wake-first condition, which was not significantly different, *t*(63.505) = -.882, *p* > .250.

## Results

Analyses were conducted on proportions of correct responses at each testing time. Data for segmentation trials (words vs. part-words) and generalisation trials (rule-words vs. part-words) from the immediate test (i.e., before the first period of sleep or wake) were first analysed to give a measure of baseline performance for these tasks, and then data from subsequent tests were analysed in combination, to assess interactions between trial type (segmentation or generalisation) and consolidation condition (sleep-first or wake-first).

### Performance at Immediate Test

Data from the test that immediately followed familiarisation were analysed to establish whether performance was affected by time of day, and to determine the baseline level of performance before the sleep or wake experimental manipulation.

For segmentation, we conducted an ANOVA with sleep condition (sleep-first or wake-first) and set-size (3X, 6X, or 12X) as factors. There was no significant effect of sleep condition or set-size on performance, and there was no significant interaction between sleep condition *F*(1, 66) = 2.197, *p* = .143, and set-size, *F* < 1. Independent samples t-tests confirmed that there was no significant difference in performance between sleep-first and wake-first participants for the 3X, *t*(22) = -.305, *p* = .763, 6X, *t*(22) = -1.800, *p* = .086, or 12X, *t*(22) = -.408, *p* = .687, conditions—indicating there were no time of day effects on initial performance.

**Fig 2:**
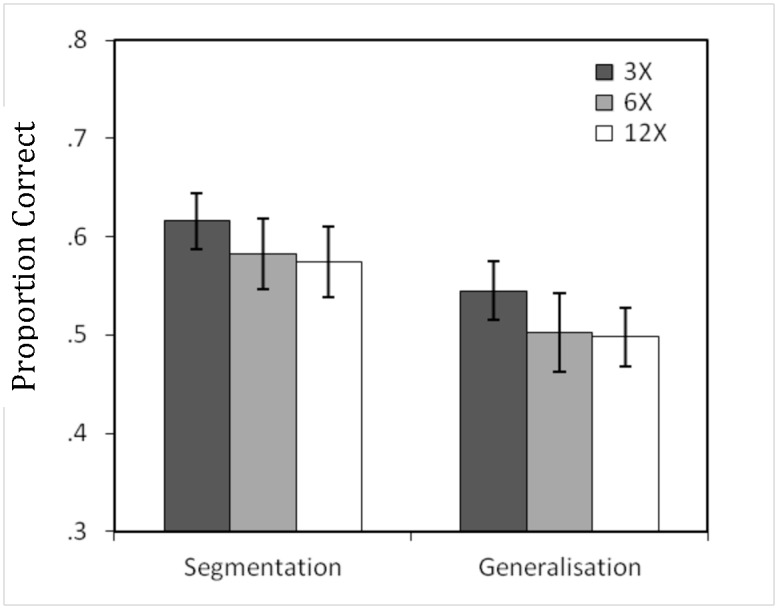
Mean proportion of correct responses on the segmentation and generalisation tasks after familiarisation for each set-size of intermediate syllables (raw accuracy scores). Error bars show standard errors.

A series of one-sample *t*-tests comparing performance to chance was conducted on the training scores of participants in each language condition (see Fig 2). Participants in all set-sizes significantly preferred words to part-words, for the 3X condition (M = .617, SE = .029), *t*(23) = 4.043, *p* = .001, *d* = 1.687, for 6X (M = .582, SE = .036, *t*(23) = 2.256, *p* = .034, *d* = .941, and for 12X (M = .574, SE = .036), *t*(23) = 2.077, *p* = .049, *d* = .866. Thus, performance on the segmentation task was higher than chance for all set sizes, showing Peña et al.’s segmentation study [12] was generalisable to variations to the stimuli.

For the grammatical generalisation task, an ANOVA with sleep condition and set-size as factors resulted in no significant effect of either variable, and no significant interaction between sleep condition and set-size, all *F* < 1. There were no significant differences in performance between sleep-first and wake-first participants for the 3X, *t*(22) = -1.327, *p* = .198, 6X, *t*(22) = —.305, *p* = .763, or 12X conditions, *t*(22) = .373 *p* = .713, so there were no distinct generalisation effects according to time of day.

A series of one-sample t-tests comparing performance to chance demonstrated no significant preference for rule-words over part-words for the 3X (M = .545, SE = .030, *t*(23) = 1.484, *p* = .151), 6X (M = .502, SE = .040, *t*(23) = .052, *p* = .959), or 12X conditions (M = .498, SE = .030, *t*(23) = -.076, *p* = .940). The lack of grammatical generalisation found in Peña et al.’s study [12] was thus replicated, and extended to languages varying in the number of X syllables intervening the non-adjacent dependencies.

As there were no effects of sleep condition in the first session, we were confident that time of day had not affected performance during familiarisation and during the immediate test. Thus, we were able to use the scores obtained during this immediate test as a baseline measure of ability. To assess our hypotheses regarding sleep-related improvement on the segmentation and generalisation tasks, the key analysis is how much individual participants’ response patterns changed as a consequence of intervals containing sleep or wake. We thus calculated participants’ improvement across experimental sessions by subtracting segmentation and generalisation scores from those obtained in the immediate test. Positive scores indicate improvement. As variability was not found to affect performance at the immediate test (and nor was it found to affect overall improvement scores), it was not included as a factor in subsequent analyses.

### Performance at Subsequent Tests

**Fig 3:**
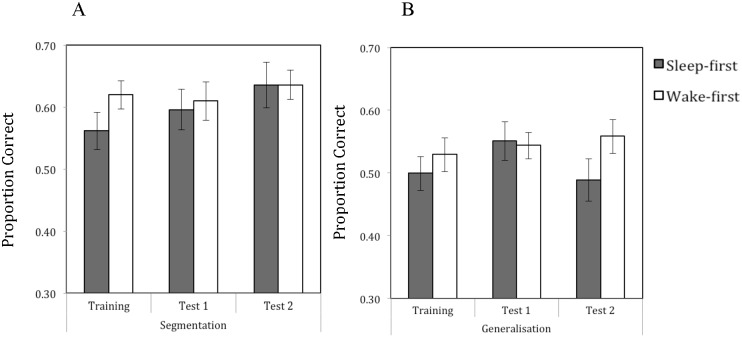
Mean performance scores on the segmentation (panel A) and generalisation (panel B) tasks immediately after training, and at each subsequent test phase, given for each consolidation condition. Error bars indicate Standard Errors.

To explore overall group effects, one sample t-tests were conducted on the data for segmentation and generalisation trials at each of the subsequent testing phases to compare performance to chance (see Fig 3). For segmentation, performance was significantly above chance for both groups at test 1 (*sleep-first*: M = .596, SE = .033, *t*(35) = 2.904, *p* = .006, *wake-first*: M = .610, SE = .031, *t*(35) = 3.616, *p* = .001) and at test 2 (*sleep-first*: M = .636, SE = .037, *t*(35) = 3.725, *p* = .001, *wake-first*: M = .636, SE = .024, *t*(35) = 5.607, *p* < .001). For generalisation, at test 1 performance was at chance level for the sleep-first group (M = .551, SE = .031, *t*(35) = 1.602, *p* = .118) and was significantly above chance for the wake-first group (M = .544, SE = .021, *t*(35) = 2.070, *p* = .046). At test 2, performance was at chance level for the sleep-first group, (M = .489, SE = .034, *t*(35) = -.325, *p* = .747), but was significantly above chance for the wake-first group (M = .559, SE = .027, *t*(35) = 2.152, *p* = .038).

### Improvement at Subsequent Tests

The critical indicator of whether these tasks were affected by sleep- or wake-related consolidation is the improvement seen between training and each of the test phases; specifically, whether this improvement showed different trajectories for each sleep group. This is because the improvement scores enable us to detect whether participants’ individual scores are systematically changing from their initial ability to perform each task.

To determine whether sleep affected segmentation and generalisation differently over the subsequent tests, we conducted a 2x2x2 repeated-measures ANOVA on the improvement scores, with test type (segmentation and generalisation), sleep group (sleep-first and wake-first) and test phase (test 1 and test 2) as factors. Crucially, there was no significant main effect of test phase, *F* < 1, meaning that overall performance was not affected by repeated testing—therefore any observed effects are due to the consolidation manipulation. Further, there was no significant main effect of sleep group, *F*(1, 70) = 1.225, *p* = .272, meaning that overall results were not driven by one particular consolidation condition. There was also no significant interaction between test phase and sleep group, *F*(1, 70) = 1.871, *p* = .176, indicating no general difference between the sleep groups at each testing phase, so any sleep effects revealed in subsequent analyses would be task-specific.

**Fig 4:**
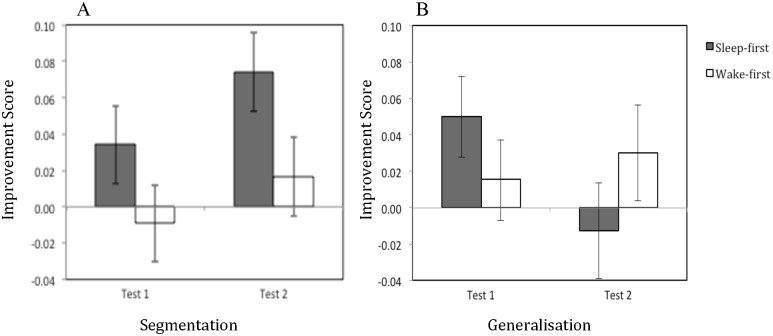
Mean improvement scores for the segmentation (panel A) and generalisation (panel B) tasks at each test phase, given for each condition. Error bars show Standard Error.

There was no significant main effect of test type, *F* < 1, but there was a significant interaction between test type and test time, *F*(1, 70) = 6.737, *p* = .011, η_p_^2^ = .088, indicating that improvement had a different time course for each of the tasks, with participants demonstrating more improvement on the generalisation task at time 1 (*segmentation*: M = .012, SE = .015, *generalisation*: M = .033, SE = .016) and more improvement on the segmentation task at time 2 (*segmentation*: M = .045, SE = .016, *generalisation*: M = .008, SE = .019). Critically, there was a significant three-way interaction between test phase, sleep group, and test type, *F*(1, 70) = 4.413, *p* = .039, η_p_^2^ = .059, indicating that participants in the sleep-first and wake-first groups demonstrated differential improvement on segmentation and generalisation trials at different stages of the experiment, due to the time in the study at which they slept (see Fig 4).

To unpack this, we analysed data for segmentation and generalisation separately using a 2x2 ANOVA, with sleep group (sleep-first and wake-first) and test phase (test 1 and test 2) as factors. For segmentation, there was a significant main effect of test phase, *F*(1, 70) = 5.489, *p* = .022, η_p_^2^ = .073, with greater improvement at test 2 (M = .045, SE = .016), than at test 1 (M = .012, SE = .015). The main effect of sleep group was approaching significance, *F*(1, 70) = 3.572, *p* = .063, η_p_^2^ = .049, with greater mean improvement for the sleep-first condition (M = .054, SE = .020) than the wake-first condition (M = .004, SE = .018) indicating that sleep soon after first exposure to the stimuli resulted in better overall performance. There was no significant interaction between test phase and sleep group, *F* < 1, due to sleep-first participants demonstrating more improvement than wake-first participants at both phases (*test 1*, *sleep-first*: M = .034, SE = .021, *wake-first*: M = -.009, SE = .021; *test 2*, *sleep-first*: M = .074, SE = .023; *wake-first*: M = .016, SE = .020. This difference was not significant at test 1, *t*(70) = 1.468, *p* = .147), but was approaching significance at test 2, *t*(70) = 1.870, *p* = .066.

For generalisation there was no significant effect of test phase, *F*(1, 70) = 2.124, *p* = .149, η_p_^2^ = .029, or sleep condition, *F* < 1. Critically though, there was a significant interaction between these two variables, *F*(1, 70) = 5.549, *p* = .021, η_p_^2^ = .073, with sleep-first participants improving more than wake-first participants at test phase 1 (*sleep-first*: M = .050, SE = .018, *wake-first*: M = .015, SE = .026) and wake-first participants improving more than sleep-first participants at test phase 2 (*sleep-first*: M = -.013, SE = .020, *wake-first*: M = .030, SE = .032), but these differences were not significant (all *p* > .250).

To further establish whether performance changes were related for the segmentation and generalisation tasks, we correlated change scores across the tasks at test phase 1 and at test phase 2. Correlations were small and not significant (*test phase 1*: *r*(70) = .083, *p* = .488, *test phase 2*: *r*(70) = .024, *p* = .842, providing no evidence that learning occurred to a similar degree on the same timescale across tasks. Correlations for sleep-first and wake-first conditions analysed separately were not distinctly different than the general correlations. Thus, there was no evidence that learning of lexical items facilitated or interfered with learning to generalise the abstract structure of the sequences—consolidation effects were isolated for each of these processes.

## Discussion

Speech segmentation and generalisation of language structure are two critically important tasks for language learning. Studies of non-adjacent dependency learning have demonstrated that learners can perform these tasks simultaneously when the non-adjacencies contain new (rather than re-positioned) intervening items [8]—suggesting that segmentation and generalisation may be governed by the same type of statistical process, contrary to prior suggestion [11, 12]. Yet, the paradigm utilised by Peña et al. in their landmark paper [12] imposes an intriguing challenge upon the learner: performing two potentially conflicting tasks from the same input. Adopting this paradigm allows us to test for the first time whether the conflicting requirements of the segmentation and generalisation task are differentially affected by sleep, and over what time course, with the possibility that sleep could enable success in both tasks without interference occurring between their respective requirements.

Our results indicate that learners were better able to segment and generalise the non-adjacent dependencies after a delay that involved a period of sleep. There are two principal implications of these findings. First, since both tasks are seen to improve, and since improvement on the generalisation task occurs in the absence of changes to the input, our findings indicate that segmentation and generalisation of non-adjacencies are not dependent upon different sets of cues, or distinct methods for parsing the input. Past research has shown that when there is conflict between memory for individual items and generalisation of structure, generalisation only occurs by changing the signal through alterations to the surface form of the stimuli [12]. These changes were suggested to increase the saliency of the respective positions of syllables [15], and facilitate the distinct computations that were previously held to be required for generalisation [12]. Since the generalisation improvement seen here occurred without changes to the input but rather as a consequence of sleep, our findings support the possibility that segmentation and generalisation of distributionally defined structures can rely on simultaneous processing of the same structural input [5, 6, 8].

The second principal implication of our findings is that sleep may help learners to isolate processing of conflicting statistical language learning tasks more effectively than an equivalent period of wakefulness. Critically, our results show that sleep-related computations assisted both segmentation and generalisation of distributionally-defined structures, but had distinct signatures on learning. The data indicate that the benefit of sleep is more pronounced for segmentation than for generalisation, supporting previous demonstrations that sleep-related consolidation is especially advantageous for learning specific instances of linguistic information [31, 32, 35]. Although effects were demonstrably more subtle than for lexical learning tasks that use pre-segmented speech, sleep was also shown to improve generalisation to a greater degree than wakefulness (evidenced by differences in improvement scores, as shown in Fig 4), in line with accounts suggesting that sleep-related learning is helpful for abstracting information relating to structure [27, 28, 34, 40, 41].

In addition to the differential effects of sleep-driven segmentation and generalisation, there is also an indication that the advantage of sleep for learning undergoes a different time-course for segmentation and generalisation. For segmentation, the benefit of sleep is evident immediately upon waking and endures over time, with results indicating an advantage for learning over 12 hours after the sleep had ended. In contrast, while the benefit of sleep for generalisation also emerges immediately, it dissipates shortly thereafter—with no such advantage at later stages of testing. It is possible that sleep promoted temporary flexible use of non-adjacencies, but long-term gains to memory for the specific pieces of information contained therein. It is worth noting that both the segmentation and generalisation task can be accomplished by extracting the rules of the stimuli, however the different trajectories of learning for segmentation and generalisation indicate that the same rule-based approach is not applied to each of these tasks. Sleep thus seems to affect processing of previously experienced instances and generalising over these instances in distinct ways.

With regard to segmentation, the improvements seen for the sleep-first group at both subsequent test phases could be seen to indicate that the benefit of sleep emerged gradually over time. A possible explanation for this is that sleep provided a protective benefit to the specific information contained within the word sequences, which reduced interference-related decay during wake [42, 43, 44, 45, 46], leading to further wakeful consolidation. Further support for this idea lies in the absolute strength of the sleep effects for each of the sleep groups: sleeping immediately after exposure to the speech resulted in greater improvement to learning than when there was a delay between initial exposure and sleep. This is in line with previous literature suggesting that immediate rather than delayed sleep typically results in most improvement to memory [33]. In our study, we have provided evidence that this also pertains to segmentation of non-adjacencies from continuous speech.

This simultaneous learning of conflicting tasks counteracts previously observed trade-offs between learning instances and learning generalisations [47, 48]. For example, in a study using a language similar to that tested here (but without a sleep manipulation), Endress and Bonatti [17] found that intensive exposure to the stimuli resulted in an increased identification of specific words, but a decrease in flexible generalisation of the sequences. Sleep could thus support improved learning of individual items without this decrement in flexibility.

Since both tasks were found to improve differentially with sleep, our pattern of results is consistent with a view that memory for words and grammar are separably served by declarative and procedural systems [49], which may be underwritten by distinct sleep-based mechanisms associated with slow-wave sleep and REM sleep, respectively. For consolidation of declarative knowledge, slow wave sleep (SWS) has been found to be particularly important, whereas for learning procedural skills, REM sleep appears to be key [20]. Gaskell et al. [45] have shown that SWS relates to improved acquisition for learning new phonotactic constraints, whereas it is the interaction between SWS and REM sleep that improves abstraction of linguistic regularities [34]. Together with previous findings of differential effects of sleep on language learning [27, 28], this indicates the possibility that learners performing the segmentation and generalisation tasks from Peña et al.’s study [12] may be able to extract the trisyllabic sequences in the language as words, *and* abstract away from the structure of those sequences as a consequence of separable sleep-related learning processes—thereby avoiding interference between these tasks.

Such a separation of sleep-related learning enables encoding of specific instances and abstraction across these instances to proceed in parallel. Our results show that sleep helps the learner to accomplish both of these tasks, and does so on different time scales. Future research testing this using polysomnography would provide conclusive evidence that this is indeed the case. For now, our results point to the possibility that these two language processing tasks can be improved simultaneously, through internal changes to the learner as a consequence of sleep.

## References

[1] Christiansen M. H. & Chater N. (2016). The Now-or-Never bottleneck: A fundamental constraint on language. *Behavioral and Brain Sciences*, 39. 10.1017/S0140525X1500031X 25869618

[2] Gerken L. A. (2006). Decisions, decisions: infant language learning when multiple generalisations are possible. *Cognition*, 98, B67–B7. 10.1016/j.cognition.2005.03.003 15992791

[3] Lany J. & Gómez R. L., & Gerken L. (2007). The role of prior experience in language acquisition, *Cognitive Science*, 31, 481–507. 10.1080/15326900701326584 21635305

[4] Pelucchi B., Hay J. F., & Saffran J. R. (2009). Statistical learning in a natural language by 8-month-old infants. *Child Development*, 80(3), 674–685. 10.1111/j.1467-8624.2009.01290.x 19489896 PMC3883431

[5] Ambridge B., & Lieven E. V. (2011). *Child language acquisition*: *Contrasting theoretical approaches*. Cambridge, UK: Cambridge University Press.

[6] Romberg A. R., & Saffran J. R. (2010). Statistical learning and language acquisition. *WileyInterdisciplinary Reviews*: *Cognitive Science*, 1(6), 906–914. 10.1002/wcs.78 21666883 PMC3112001

[7] Altmann G. T. M. (2002). Statistical learning in infants. *Proceedings of the National Academy of Sciences*, *USA*, 99(24), 15250–15251. 10.1073/pnas.262659399 PMC137701 12438680

[8] Frost R. L. A., & Monaghan P. (2016). Simultaneous segmentation and generalisation of non-adjacent dependencies from continuous speech. *Cognition*, 147, 70–74. 10.1016/j.cognition.2015.11.010 26638049

[9] van den Bos E., Christiansen M. H., & Misyak J. B. (2012). Statistical learning of probabilistic nonadjacent dependencies by multiple-cue integration. *Journal of Memory and Language*, 67(4), 507–520.

[10] Chomsky N. (2005). Three factors in language design. *Linguistic Inquiry*, 36, 1–22.

[11] Marcus G. F., Vijayan S., Rao S. B., & Vishton P. M. (1999). Rule learning by seven month-old infants. *Science*, 283, 77–80. 9872745 10.1126/science.283.5398.77

[12] Peña M., Bonatti L., Nespor M., & Mehler J. (2002). Signal-driven computations in speech processing. *Science*, 298, 604–607. 10.1126/science.1072901 12202684

[13] Tomasello M. (2003). *Constructing a language*: *A usage-based theory of language acquisition*. Cambridge, MA: Harvard University Press.

[14] Ambridge B. (2013). How do children restrict their linguistic generalizations? An (un-)grammaticality judgment study. *Cognitive Science*, 37(3), 508–543. 10.1111/cogs.12018 23252958 PMC3644877

[15] Perruchet P., Tyler M. D., Galland N., & Peereman R. (2004). Learning non-adjacent dependencies: No need for algebraic-like computations. *Journal of Experimental Psychology*: *General*, 133(4), 573–583. 15584807 10.1037/0096-3445.133.4.573

[16] Perruchet P., Peereman R., & Tyler M. D. (2006). Do we need algebraic-like computations? A reply to Bonatti, Peña, Nespor, and Mehler. *Journal of Experimental Psychology*, 135(2), 322–326.

[17] Endress A. D., & Bonatti L. L. (2007). Rapid learning of syllable classes from a perceptually continuous speech stream. *Cognition*, 105(2), 247–299. 10.1016/j.cognition.2006.09.010 17083927

[18] Mueller J. L., Bahlmann J., & Friederici A. D. (2008). The role of pause cues in language learning: The emergence of event-related potentials related to sequence processing. *Journal of Cognitive Neuroscience*, 20(5), 892–905. 10.1162/jocn.2008.20511 18201121

[19] Kumaran D. & McClelland J. L. (2012). Generalization through the recurrent interaction of episodic memories: A model of the hippocampal system. *Psychological Review*, 119, 573–616. 10.1037/a0028681 22775499 PMC3444305

[20] Rasch B. & Born J. (2013). About sleep's role in memory. *Physiological Reviews* 93, 681–766. 10.1152/physrev.00032.2012 23589831 PMC3768102

[21] Fenn K., Nusbaum H., & Margoliash D. (2003). Consolidation during sleep of perceptual learning of spoken language. *Nature*, 425, 614–616. 10.1038/nature01951 14534586

[22] Mednick S., Nakayama K., & Stickgold R. (2003). Sleep-dependent learning: A nap is as good as a night. *Nature Neuroscience*, 6(7), 697–698. 10.1038/nn1078 12819785

[23] Wagner U., Gais S., Haider H., Verleger R., & Born J. (2004). Sleep inspires insight. *Nature*, 427, 352–355. 10.1038/nature02223 14737168

[24] Gais S. & Born J. (2004). Low acetylcholine during slow-wave sleep is critical for declarative memory consolidation. *Proceedings of the National Academy of Sciences*, 101, 2140–2144. 10.1073/pnas.0305404101 PMC357065 14766981

[25] Hasselmo M. E. (1999). Neuromodulation: acetylcholine and memory consolidation. *Trends in Cognitive Sciences*, 3, 351–359. 10461198 10.1016/s1364-6613(99)01365-0

[26] Hasselmo M. E. & McGaughy J. (2004). High acetylcholine levels set circuit dynamics for attention and encoding and low acetylcholine levels set dynamics for consolidation. *Progress in Brain Research*, 145, 207–231. 10.1016/S0079-6123(03)45015-2 14650918

[27] Nieuwenhuis I. L. C., Folia V., Forkstam C, Jensen O. & Petersson K. M. (2013). Sleep promotes the extraction of grammatical rules. *PLoS ONE* 8(6): E65046 10.1371/journal.pone.0065046 23755173 PMC3673983

[28] Gómez R. L., Bootzin R. R., & Nadel L. (2006). Naps promote abstraction in language learning infants. *Psychological Science*, 17, 670–674. 10.1111/j.1467-9280.2006.01764.x 16913948

[29] Mirković J., & Gaskell M.G. (2016). Does sleep improve your grammar? Preferential consolidation of arbitrary components of new linguistic knowledge. *PLoS ONE*, 11(4), e0152489 10.1371/journal.pone.0152489 27046022 PMC4821602

[30] Brown H., Weighall A., Henderson L. M., & Gaskell M. G. (2012). Enhanced recognition and recall of new words in 7- and 12-year old children following a period of offline consolidation. *Journal of Experimental Child Psychology*, 112, 56–72. 10.1016/j.jecp.2011.11.010 22244988

[31] Dumay N., & Gaskell M. G. (2005). Do words go to sleep? Exploring consolidation of spoken forms through direct and indirect measures. *Behavioral and Brain Sciences*, 28, 69–70.

[32] Dumay N., & Gaskell M. G. (2007). Sleep-associated changes in the mental representation of spoken words. *Psychological Science*, 18, 35–39. 10.1111/j.1467-9280.2007.01845.x 17362375

[33] Gais S., Lucas B., & Born J. (2006). Sleep after learning aids memory recall. *Learning and Memory*, 13, 259–262. 10.1101/lm.132106 16741280 PMC10807868

[34] Batterink L.J., Oudiette D., Reber P.J., & Paller K. A. (2014). Sleep facilitates learning a new linguistic rule. *Neuropsychologia*, 65, 169–179. 10.1016/j.neuropsychologia.2014.10.024 25447376 PMC4259849

[35] Williams S. E. & Horst J. S. (2014). Goodnight book: sleep consolidation improves word learning via storybooks. *Frontiers in Psychology*, 5, 184 10.3389/fpsyg.2014.00184 24624111 PMC3941071

[36] Morgan, K., David, B., & Gascoigne, C. (2007). NHS Sleep Diary, Clinical Sleep Research Unit Loughborough University UK.

[37] Black, A. W., Taylor, P., & Caley, R. (1990). The Festival Speech Synthesis System, http://www.cstr.ed.ac.uk/projects/festival.html, Centre for Speech Technology Research (CSTR), University of Edinburgh, Edinburgh, UK.

[38] Gómez R. L. (2002). Variability and detection of invariant structure, *Psychological Science*, 13, 431–436. 12219809 10.1111/1467-9280.00476

[39] Sadeh A., Sharkey K. M., & Carskadon M. A. (1994). Activity-based sleep-wake identification: An empirical test of methodological issues. *Sleep*, 17, 201–207. 7939118 10.1093/sleep/17.3.201

[40] Lewis P. A. & Durrant S. J. (2011). Overlapping memory replay during sleep builds cognitive schemata. *Trends in Cognitive Sciences*, 15, 343–351. 10.1016/j.tics.2011.06.004 21764357

[41] Stickgold R., Walker M.P. 2013 Sleep-dependent memory triage: evolving generalization through selective processing. *Nat*. *Neurosci*. 16, 139–145. 10.1038/nn.3303 23354387 PMC5826623

[42] Alger S. E., Lau H., & Fishbein W. (2012). Slow wave sleep during a daytime nap is necessary for protection from subsequent interference and long-term retention. *Neurobiology of Learning and Memory*, 98, 188–196. 10.1016/j.nlm.2012.06.003 22732649

[43] Diekelmann S., Biggel S., Rasch B., & Born J. (2012). Offline consolidation of memory varies with time in slow wave sleep and can be accelerated by cuing memory reactivations. *Neurobiology of Learning and Memory*, 98(2):103–11. 10.1016/j.nlm.2012.07.002 22789831

[44] Ellenbogen J. M, Hulbert J. C., Jiang Y., & Stickgold R. (2009). The sleeping brain's influence on verbal memory: boosting resistance to interference. *PLoS ONE*, 4(1), E4117 10.1371/journal.pone.0004117 19127287 PMC2606059

[45] Gaskell M. G., Warker J., Lindsay S., Frost R., Guest J., Snowdon R. & Stackhouse A. (2014). Sleep underpins the plasticity of language production. *Psychological Science*, 25, 1457–1465. 10.1177/0956797614535937 24894583

[46] Sheth B. R., Varghese R. & Truong T. (2012). Sleep shelters verbal memory from different kinds of interference. *Sleep*, 35(7), 985–996. 10.5665/sleep.1966 22754045 PMC3369234

[47] Tamminen J., Davis M. H., Merkx M. & Rastle K. (2012). The role of memory consolidation in generalisation of new linguistic information. *Cognition*, 125, 107–112. 10.1016/j.cognition.2012.06.014 22832178

[48] Lau H., Alger SE, Fishbein W (2011) Relational Memory: A daytime nap facilitates the abstraction of general concepts. *PLoS ONE* 6(11): e27139 10.1371/journal.pone.0027139 22110606 PMC3217953

[49] Ullman M. T. (2004). Contributions of memory circuits to language: The declarative/ procedural model. *Cognition*, 92, 231–270. 10.1016/j.cognition.2003.10.008 15037131

